# Investigation of serum Metrnl levels in pregnant women with gestational diabetes mellitus: a prospective non-interventional cohort study

**DOI:** 10.1590/1806-9282.20240660

**Published:** 2024-10-07

**Authors:** Sabina Damirova, İbrahim Kale, Ayşegül Özel, Ayşe Keleş, Cem Yalçınkaya, Murat Muhcu

**Affiliations:** 1 Umraniye Training and Research Hospital, Department of Obstetrics and Gynecology – İstanbul, Turkey.; 2 Umraniye Training and Research Hospital, Department of Obstetrics and Gynecology, Maternal Fetal Unit – İstanbul, Turkey.

**Keywords:** Gestational diabetes mellitus, Metrnl

## Abstract

**OBJECTIVE:**

The objective of this study was to investigate serum Metrnl levels in pregnant women with gestational diabetes mellitus and compare them with pregnant women without gestational diabetes mellitus.

**METHODS:**

The gestational diabetes mellitus group consisted of 87 pregnant women diagnosed with gestational diabetes mellitus, and the control group consisted of 93 healthy pregnant women without gestational diabetes mellitus. Serum Metrnl levels were determined by the enzyme-linked immunosorbent assay method.

**RESULTS:**

The two groups were similar in terms of demographic features. The median serum Metrnl level was found to be 1.16 ng/mL in the gestational diabetes mellitus group, while it was determined as 2.2 ng/mL in the control group (p=0.001). The two groups were divided into two subgroups based on participants’ body mass index, normal weight and overweight. The lowest median Metrnl level was detected in the normal weight gestational diabetes mellitus group, followed by the overweight gestational diabetes mellitus group, normal weight control group, and overweight control group (1.1, 1.2, 2, and 2.4 ng/mL, respectively). Receiver operating curve analysis was performed to determine the value of the serum Metrnl level in terms of predicting gestational diabetes mellitus. The area under the curve analysis of serum Metrnl for gestational diabetes mellitus estimation was 0.768 (p=0.000, 95%CI 0.698–0.839). The optimal cutoff value for serum Metrnl level was determined as 1.53 ng/mL with 69% sensitivity and 70% specificity.

**CONCLUSION:**

Serum Metrnl levels in pregnant women with gestational diabetes mellitus were found to be significantly lower than in pregnant women without gestational diabetes mellitus. The mechanisms underlying the decrease in serum Metrnl levels in gestational diabetes mellitus remain unclear for now, and future studies will reveal the role of Metrnl in the pathophysiology of gestational diabetes mellitus.

## INTRODUCTION

Gestational diabetes mellitus (GDM) is considered abnormal glucose tolerance that begins during pregnancy or is detected for the first time during pregnancy^1^. As the pregnancy progresses, significant changes occur in carbohydrate metabolism to meet the needs of the growing fetus^2^. Placenta-related hormones such as corticotropin-releasing hormone, human placental lactogen, prolactin, estrogen, and progesterone contribute to the development of insulin resistance^1^. Excess weight gained and increased fat tissue also contribute to insulin resistance^3^.

It has been shown in animal studies that increased insulin secretion to maintain euglycemia during pregnancy is achieved by hypertrophy and hyperplasia of β cells in the pancreas^4^. However, in some pregnant women, euglycemia cannot be sustained as a result of insufficient amount of insulin secreted from the pancreas or insulin resistance caused by interactions at the receptor level^1,5^. As a result, GDM occurs in some pregnant women with a possible genetic and epigenetic background, the effect of placental hormones, and the contribution of nutrition^1^.

There is no clear consensus on a single screening tool for screening for preexisting diabetes in early pregnancy. Fasting blood glucose, HbA1c, and the 75 g 2-h oral glucose tolerance test (OGTT) are currently used to screen for preexisting DM in early pregnancy^6^. For GDM screening in the later weeks of pregnancy, the International Diabetes in Pregnancy Working Groups (IADPSG) and the World Health Organization (WHO) approved the 2-h 75 g single-step OGTT^7,8^.

In 2017, a meta-analysis was published that included 7 studies involving 1,420 women, examining different strategies for diagnosing GDM to improve maternal and infant health. The following six comparisons were evaluated in this meta-analysis: 100 g OGTT versus 75 g OGTT, 50 g glucose monomer drink versus candy bar, 50 g glucose monomer drink versus 50 g glucose polymer drink, 50 g glucose food versus 50 g glucose drink, 75 g glucose g OGTT WHO criteria versus 75 g OGTT American Diabetes Association criteria, and two-step approach versus one-step approach. The authors concluded that there is insufficient evidence to recommend which strategy is best for diagnosing GDM. They noted that large randomized studies are needed to establish the best strategy for accurately identifying women with GDM^9^.

An important problem in GDM screening is that some pregnant women refuse to have an OGTT for various reasons. In a paper from our country, the rate of those who had OGTT for GDM screening was reported as 79.4%. The most common reason why pregnant women refuse OGTT is that they think OGTT is unnecessary or the belief that OGTT will be harmful to the fetus^10^.

Currently, there is no single protocol accepted worldwide for the definition of the population to be screened for GDM, the gestational week at which screening will be performed, and the test or threshold values to be used in screening. Each country or region follows the protocols it has accepted^1^. Many publications continue to be reported in the literature investigating new molecules, ultrasound measurements, or prediction models that can be used in GDM screening^11–16^.

Meteorin-like protein, also known as Metrnl, Meteorin-b, Cometin, and Subfatin, exerts its pleiotropic effects on glucose metabolism, immunology, and inflammation^17^. Studies have reported that Metrnl reduces insulin resistance. A paper published in 2015 showed that Metrnl antagonized insulin resistance through peroxisome proliferator-activated receptor γ (PPARγ) signaling^18^. In 2018, it was reported that Metrnl reduces insulin resistance through AMP-activated protein kinase (AMPK) or peroxisome proliferator-activated receptor δ (PPARδ)-dependent pathways in skeletal muscle^19^. In 2021, it was shown that Metrnl ameliorates the β-cell function by inhibiting apoptosis and promoting β-cell proliferation via activating the WNT/β-catenin pathway^20^. Additionally, Yao et al. showed that the intravenous administration of Metrnl in mice suppressed autoreactive T-cell activity, reduced the severity of insulitis, and delayed the onset of diabetes in nonobese diabetic mice^21^.

Despite the insulin resistance-reducing effect of Metrnl reported in the studies mentioned above, conflicting results between serum Metrnl levels and diabetes mellitus (DM) have been reported in clinical studies. While some studies have reported that serum Metrnl levels of Type 2 DM (TP2DM) patients are lower than normoglycemic healthy controls, in some studies, high serum Metrnl levels have been associated with the development of TP2DM^22–24^.

While this uncertainty continued regarding serum Metrnl levels in individuals with type 2 diabetes mellitus (T2DM), we wondered about Metrnl levels in the serum of pregnant women with GDM and conducted this clinical study.

## METHODS

This prospective non-interventional cohort study was conducted with 180 pregnant women aged between 18 and 39 years who applied to the Umraniye Training and Research Hospital, Department of Obstetrics and Gynecology, Istanbul, Turkey, between June 2023 and October 2023. While the GDM group consisted of 87 pregnant women diagnosed with GDM, the control group consisted of 93 healthy pregnant women with normal 75-g OGTT results. GDM and control groups were matched in terms of age, body mass index (BMI), and gestational week at blood sampling for Metrnl.

Smokers, multiple pregnancies, and those who conceive with the in vitro fertilization method were not included in the study. Those with any pregestational disease or a history of GDM in previous pregnancies were not included in the study. Those who developed any pregnancy-related disease other than GDM in the GDM group and those who developed any pregnancy-related disease in the control group were not included in the study.

The 75 g OGTT was applied to all participants between 24 and 28 weeks of gestation. OGTT results were evaluated according to the criteria recommended by the IADPSG^7^.

To investigate serum Metrnl levels, peripheric blood samples were taken from participants after an 8-h fast in the morning. The blood samples were centrifuged at 4,000 rpm for 10 min. After centrifugation, the remaining serum in the upper part of the biochemistry tube was transferred to the Eppendorf tube and stored at −80 degrees. Metrnl levels were studied with the human Meteorin-like protein (Metrnl) enzyme-linked immunosorbent assay (ELISA) kit (Sunredbio, Catalog No: 201-12-9252) using the ELISA method. For the human Meteorin-like protein ELISA kit used in the study, a measurement value of 0.05–15 ng/mL and a sensitivity of 0.042 ng/mL were determined.

First, the GDM group and the control group and then the subgroups were compared in terms of serum Metrnl levels.

The Istanbul Umraniye Training and Research Hospital Local Ethics Committee approved this study (Approval Number: B.10.1.TKH.4.34.H.GP.0.01/162). The study was conducted in accordance with the Declaration of Helsinki and followed the ethical standards of the country. Informed and written consent was obtained from all participants.

### Statistical analysis

Power analysis of the study was performed using the G*Power (v3.1.9) program to determine sample sizes. The power of the study is expressed as 1–β (β=Type II error probability) and has 99% power. Assuming that the effect size (d=0.5) will be observed according to the effect size coefficients determined by Cohen, it was determined that the required number of patients should be 160 (80 for the GDM group and 80 for the control group). Considering the dropouts during the study, 95 participants were included in each group. After dropouts, the study was conducted on a total of 180 participants, with 87 in the GDM group and 93 in the control group.

Statistical analysis was performed with the Statistical Package for the Social Sciences version 25.0. The Kolmogorov-Smirnov test was used to determine whether the data were distributed normally or not. Descriptive statistical methods (mean, standard deviation, median, Min, Max, frequency, and rate) were used while evaluating the study data. An Independent t-test was used for the comparison of two groups showing parametric distribution, and the Mann-Whitney U test was used for the comparison of two groups showing nonparametric distribution. The chi-square test was used in the analysis of categorical data. The one-way analysis of variance (ANOVA) test was used for the comparison of more than two groups showing parametric distribution, and the Kruskal-Wallis test was used for the comparison of more than two groups showing nonparametric distribution. The receiver operating curve (ROC) was used to determine the significant threshold of Metrnl in predicting GDM. Statistical significance was accepted at p<0.05 for all values.

## RESULTS

Both groups were similar in terms of age, parity, BMI at blood sampling, and gestational week at blood sampling (p>0.005, for all). Fasting, first-hour, and second-hour glucose levels in the OGTT were significantly higher in the GDM group than in the control group (p=0.001, for all). Gestational week at delivery, birth weight, first-minute Apgar score, fifth-minute Apgar score, and neonatal intensive care unit admission were similar in both groups (p<0.05, for all). The median serum Metrnl level was found to be 1.16 ng/mL in the GDM group, while it was determined as 2.2 ng/mL in the control group (p=0.001) (Table 1).

**Table 1:** Comparison of gestational diabetes mellitus and control groups in terms of demographic characteristics, laboratory findings, and perinatal outcomes.

		Control group (n=93)	GDM group (n=87)	p-value
		Mean±SD Median (min–max) n (%)	Mean±SD Median (min–max) n (%)	
Age (years)		28.72±4.4 29 (19–39)	29.8±4.4 30 (20–39)	0.076*
Parity	Nulliparous	32 (34.4)	32 (36.8)	0.740**
	Multiparous	61 (65.6)	55 (63.2)	
BMI at blood sampling (kg/m^2^)		27.9±5.0 25.9 (19.3–47.8)	28.1±3.9 28 (21.2–36.6)	0.212*
Gestational week at blood sampling		25.7±1.3 26 (24–28)	26±1.6 26 (24–28)	0.311*
75-g OGTT fasting blood glucose level (mg/dL)		79.4±7.4 79 (65–91)	92.2±12.3 93 (65–138)	0.001*
75-g OGTT first-hour blood glucose level (mg/dL)		123.1±23.7 126 (74–173)	180.8±30.4 185 (93–247)	0.001*
75-g OGTT second-hour blood glucose level (mg/dL)		104.8±20.3 106 (48–152)	142.9±31.3 145 (80–255)	0.001*
Serum Metrnl (ng/mL)		3.23±2.7 2.2 (0.6–13.2)	1.73±1.47 1.16 (0.55–7.63)	0.001*
Gestational week at delivery		38.1±1 38 (37–41)	38±1.1 38 (37–41)	0.567*
Birth weight (g)		3,318±306 3,310 (2,847–4,150)	3,353±337 3,380 (2,810–4,215)	0.568*
First-minute Apgar score		7.8±0.8 8 (6–9)	7.8±1 8 (5–9)	0.767*
Fifth-minute Apgar score		8.9±0.7 9 (7–10)	8.7±0.8 9 (6–10)	0.137*
NICU admission		36 (38.7)	39 (44.8)	0.405**

* Mann-Whitney U test;

** chi-square test;

BMI: body mass index; NICU: neonatal intensive care unit; GDM: gestational diabetes mellitus; OGTT: oral glucose tolerance test.

The participants were divided into two subgroups according to their BMI. Those with a BMI below 25 kg/m^2^ were classified as normal weight, and those with a BMI of 25 kg/m^2^ and above were classified as overweight. Age and gestational week at blood sampling were similar in these four groups (p<0.05, for all). The lowest median Metrnl level was detected in the normal weight GDM group, followed by the overweight GDM group, normal weight control group, and overweight control group (1.1, 1.2, 2, and 2.4 ng/mL, respectively) (Table 2).

**Table 2:** Comparison of the control group with normal weight, control group with overweight, gestational diabetes mellitus group with normal weight, and gestational diabetes mellitus group with overweight in terms of serum Metrnl concentrations.

	Control group normal weight (n=32)	Control group overweight (n=61)	GDM group normal weight (n=29)	GDM group overweight (n=58)	p-value	Post hoc
	Mean±SD Median (min–max)	Mean±SD Median (min–max)	Mean±SD Median (min–max)	Mean±SD Median (min–max)		
Age (years)	28±4.6 28.5 (19–37)	29±4.3 29 (21–39)	29±3.7 30 (20–35)	30.3±4.5 30 (21–39)	0.219	
BMI at blood sampling (kg/m^2^)	22.8±1.4 23.2 (19.3–24.8)	29.5±4.6 28.6 (25–47.8)	23.9±1.2 24.5 (21.2–24.9)	30.2±2.9 29.2 (25.9–36.6)	<0.001	2>1 (<0.001) 3>1 (0.027) 4>1 (<0.001) 2>3 (<0.001) 4>3 (<0.001)
Gestational week at blood sampling	25.7±1.2 25.5 (24–28)	25.7±1.3 26 (24–28)	26.4±1.5 26 (24–28)	25.8±1.6 26 (24–28)	0.803	
Serum Metrnl (ng/mL)	2.8±2.08 2 (0.6–9)	3.44±2.97 2.4 (0.8–13.2)	1.68±1.51 1.1 (0.5–6.8)	1.76±1.47 1.2 (0.6–7.6)	0.001	2>3 (0.002) 2>4 (0.001)

Kruskal-Wallis test; BMI: body mass index.

**Figure 1:**
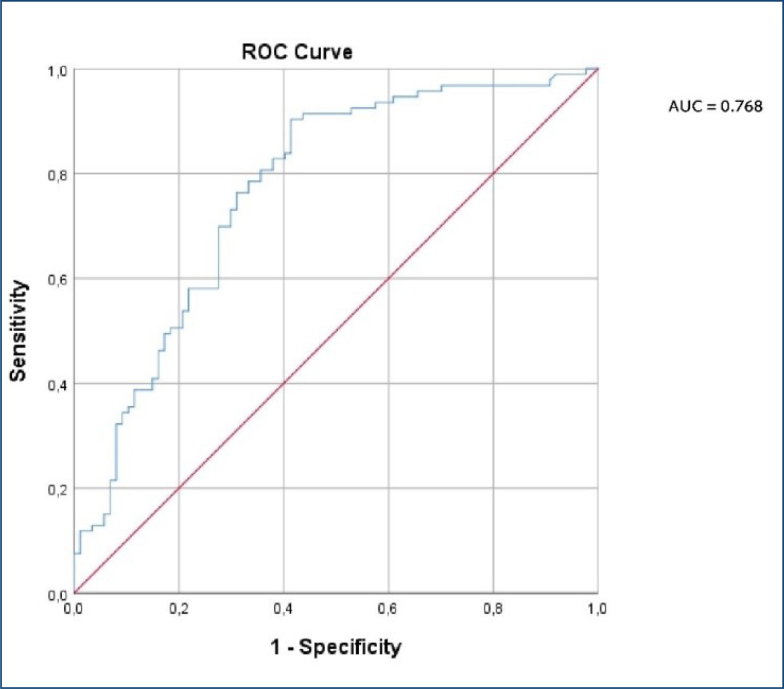
Receiver operating curve analysis for sensitivity, specificity, and positive and negative predictive value of serum Metrnl in gestational diabetes mellitus.

ROC analysis was performed to determine the value of the serum Metrnl level in terms of predicting GDM. The area under the curve (AUC) analysis of serum Metrnl for GDM estimation was 0.768 (p=0.000, 95%CI 0.698–0.839). The optimal cutoff value for the serum Metrnl level was determined as 1.53 ng/mL with 69% sensitivity and 70% specificity (Figure 1).

## DISCUSSION

After animal and cell culture studies revealed the effect of Metrnl in reducing insulin resistance through different pathways, clinical studies on glucose metabolism and Metrnl began to accumulate in the literature. In a study published in 2018, serum Metrnl levels were evaluated in 400 individuals with T2DM and 400 healthy individuals without DM. The serum Metrnl level was found to be significantly higher in patients with T2DM than in those without DM^25^. In another study published in 2019, 59 individuals with T2DM and 30 individuals with normal glucose tolerance (NGT) were compared in terms of serum Metrnl levels. Compared to the NGT group, the serum Metrnl level was found to be higher in the T2DM group^26^. In a different study published in 2019, serum Metrnl levels were investigated in individuals with NGT, impaired fasting glucose (IFG), impaired glucose tolerance (IGT), and newly diagnosed T2DM individuals. The highest serum Metrnl level was detected in the newly diagnosed T2DM group, followed by the IGT group, IFG group, and NGT group, respectively^23^.

Contrary to the abovementioned results, some clinical studies have reported low serum Metrnl levels in patients with diabetes. In 2020, Fadaei et al. published a study in which they investigated serum Metrnl levels in 52 T2DM patients, 39 prediabetic individuals, and 50 healthy individuals. Accordingly, the lowest serum Metrnl level was detected in the T2DM group, followed by the prediabetes group and the normal healthy control group^27^. In a different study published in 2020, serum Metrnl levels were evaluated in 50 newly diagnosed T2DM patients, 50 long-standing diagnosed T2DM patients, and 50 healthy diabetes-free individuals as a control group. The lowest serum Metrnl level was detected in the long-standing diagnosed T2DM group, followed by the newly diagnosed T2DM and the control group^28^. In 2022, Timurkaan et al. compared serum Metrnl levels in 60 individuals with T2DM and 60 healthy individuals without DM. The authors found that serum Metrnl levels in the diabetic group were significantly lower than in the control group^29^.

Similar to the contradictory results reported in studies on serum Metrnl levels in individuals with DM, conflicting results have also been reported on serum Metrnl levels in pregnant women with GDM. Yavuzkır et al. measured serum Metrnl levels in 30 pregnant women with GDM and 30 normal healthy pregnant women (as a control group) between the 24th and 28th weeks of gestation and after the delivery. Serum Metrnl levels were higher in the GDM group than in the control group, both in blood samples taken between the 24th and 28th weeks of gestation and in blood samples taken after delivery^30^. Contrary to the result reported in this study, in our study, we found serum Metrnl levels to be significantly lower in the GDM group than in the control group between 24 and 28 weeks of gestation.

In the study published by Lappas in 2021, they evaluated the effect of maternal obesity and GDM on the serum Metrnl concentration in the newborn's umbilical cord. Metrnl levels were measured on maternal serum and umbilical cord plasma from pregnant women with NGT (19 nonobese and 20 obese), GDM controlled by diet (18 nonobese and 17 obese), and GDM controlled by insulin (19 nonobese and 18 obese). Maternal serum Metrnl levels were similar in the nonobese NGT, obese NGT, diet-controlled nonobese GDM, diet-controlled obese GDM, insulin-using nonobese GDM, and insulin-using obese GDM groups. The authors suggested that maternal obesity or GDM did not affect plasma Metrnl levels^31^. Unlike the result of this study, in our study, we found that serum Metrnl levels in the overweight control group were significantly higher than the normal weight GDM group and the overweight GDM group in the subgroup analysis performed according to BMI.

Finally, in 2023, Yavuz et al. published a study examining Metrnl levels in the serum and milk of pregnant women with GDM and its expression in breast tissue at term. The authors found that Metrnl levels in both serum and milk were significantly increased in mothers with GDM compared to GDM-free controls^32^. Contrary to this result, in our study, we found that serum Metrnl levels measured between the 24th and 28th weeks of gestation in the GDM group were significantly lower than the control group.

This single-center study has important limitations. First of all, the study was conducted with a limited number of participants. Also, serum Metrnl was measured only once in participants between the 24th and 28th weeks of pregnancy. Therefore, serum Metrnl levels are single-point observations and these values will not reflect the dynamic change in serum throughout the entire pregnancy period.

In conclusion, in this study, serum Metrnl levels in pregnant women with GDM were found to be significantly lower than in pregnant women without GDM. The mechanism underlying the reduction of Metrnl levels in GDM remains unclear. Unraveling the role of Metrnl in the pathophysiology of GDM is the subject of future studies.
